# Immunofluorescence identifies distinct subsets of endothelial cells in the human liver

**DOI:** 10.1038/srep44356

**Published:** 2017-03-13

**Authors:** Otto Strauss, Anthony Phillips, Katya Ruggiero, Adam Bartlett, P. Rod Dunbar

**Affiliations:** 1 Department of Surgery, Faculty of Medical Health Sciences, University of Auckland, Auckland, New Zealand; 2 Maurice Wilkins Centre for Molecular Biodiscovery, University of Auckland, Auckland, New Zealand; 3 School of Biological Sciences, Faculty of Science, University of Auckland, Auckland, New Zealand; 4 Department of Statistics, University of Auckland, Auckland, New Zealand

## Abstract

As well as systemic vascular endothelial cells, the liver has specialised sinusoidal endothelial cells (LSEC). LSEC dysfunction has been documented in many diseased states yet their phenotype in normal human liver has not been comprehensively assessed. Our aim was to improve characterisation of subsets of endothelial cells and associated pericytes in the human liver. Immunofluorescence microscopy was performed on normal human liver tissue samples to assess endothelial and structural proteins in a minimum of three donors. LSEC are distributed in an acinar pattern and universally express CD36, but two distinctive subsets of LSEC can be identified in different acinar zones. Type 1 LSEC are CD36^hi^CD32^−^CD14^−^LYVE-1^−^ and are located in acinar zone 1 of the lobule, while Type 2 LSEC are LYVE-1^+^CD32^hi^CD14^+^CD54^+^CD36^mid-lo^ and are located in acinar zones 2 and 3 of the lobule. Portal tracts and central veins can be identified using markers for systemic vascular endothelia and pericytes, none of which are expressed by LSEC. In areas of low hydrostatic pressure LSEC are lined by stellate cells that express the pericyte marker CD146. Our findings identify distinctive populations of LSEC and distinguish these cells from adjacent stellate cells, systemic vasculature and pericytes in different zones of the liver acinus.

The liver lobule is a complex structure composed of hepatocytes and numerous non-parenchymal cells; including lymphocytes, antigen presenting cells (APC), stellate cells, endothelial cells, and liver sinusoidal endothelial cells (LSEC)^1^. The intention of this study was to define molecules differentially expressed by endothelial cells and pericytes in different zones of the human liver.

The microanatomy of liver lobule can be described by the liver acinar model. The acinar model revolves around the repeating units of lobular tissue defined by their afferent blood supply^2^. Two portal tracts (PT) each drain mixed blood from the hepatic artery and portal vein into the lobule which can be divided into 3 zones. The area between two PT is known as Zone 1 (Z1), and contains blood of the highest oxygen pressure. Zone 3 (Z3) describes the lobule nearest to the central vein (CV), and Zone 2 (Z2) refers to the area that lies between Z1 and Z3, otherwise without clear anatomical distinction^2^. Blood enters the lobule in Z1 and then percolates down towards Z3 before draining into the systemic circulation via the CV.

An understanding of the endothelial system is essential to appreciating normal physiology but also disease processes. Changes in LSEC composition have been observed in a number of conditions such as hepatic fibrosis^3^, autoimmune disease^4^, and viral hepatitis^5^. A map of the endothelial structures in the normal liver is essential to appreciate the structural and molecular changes that take place in diseases of the liver.

The acinar zones represent different microenvironments in the lobule. Hepatocytes have been shown to determine the immunophenotype of neighbouring endothelial cells^6^,^7^, and differences in molecular phenotype have been observed between periportal and pericentral hepatocytes (Z1 and Z3 respectively)^2^,^8^. In animals, LSECs differ across acinar zones, as indicated by their expression of different lectin-binding glycans^9^. In humans, some electron microscopy studies revealed structural differences between periportal and pericentral LSEC, such as the size of LSEC membrane fenestrae^10^,^11^. Taken together, all these data suggested LSEC may also be heterogeneous at the molecular level.

A number of histochemical markers have been used to distinguish LSEC from vascular endothelium^3^ including CD32 (the type II Fc gamma receptor)^12^, CD299 (also known as liver/lymph node-specific intracellular adhesion molecules-3 grabbing non-integrin - L-SIGN)^13^, and the lymphatic marker LYVE-1 (lymphatic vessel endothelial hyaluronan receptor-1)^14^. LSEC are also reported to stain for CD36^12^ but in two studies, vascular endothelia also appeared to be CD36^+^ 12,15. Most of the published single colour immunohistochemistry studies do not report any distinctive distribution of LSEC markers indicative of LSEC heterogeneity^12^,^13^,^15^. However there are some data that indicated human LSEC do indeed show molecular heterogeneity that follows an acinar distribution^14^,^16^. Scoazec *et al*. reported that while LSEC in Z2/Z3 express both CD14 and CD16 (the type III Fc gamma receptor), these markers are lacking from LSEC in Z1^16^. Mouta Carreira and colleagues noted that LYVE-1, although specific for LSECs in the liver, stain LSEC predominantly in Z2^14^.

We recently used multicolour immunofluorescence microscopy (IFM) to definitively characterise the heterogeneity of lymphatic endothelium in human lymph nodes^17^. In this study we applied similar techniques to human liver to characterise the different populations of vascular cells, and to investigate whether distinctive LSEC sub-populations are distributed in an acinar pattern.

## Materials and Methods

### Human Tissues

Human liver specimens were obtained from living donors undergoing hepatic resection (listed in Supplementary Table 1). Skin biopsy specimens that were used as positive control tissue were obtained following abdominoplasty and breast reduction surgery. All samples studied had no histological abnormality. No tissue samples were obtained from executed prisoners or other institutionalised persons. Patients gave written informed consent, and all experiments and methods were performed in accordance with relevant guidelines and regulations under protocols approved by the Northern Human Research Ethics Committee, Auckland, New Zealand (Approval reference number NTY/09/12/117 for liver tissue and Approval reference number NTX0809086 for skin tissue).

### Multicolour immunofluorescence microscopy

Liver specimens were either embedded in TissueTek OCT compound (Sakura Finetek, Zoeterwoude, The Netherlands), snap frozen in a bath of isopentane that had been chilled in liquid nitrogen and stored at −80 °C or were soaked in 10% paraformaldehyde overnight at room temperature before being embedded in paraffin at 37 °C. Sections 3–5 μm thick were cut using either a cryotome for frozen tissue, or a microtome for multicolour immunofluorescence microscopy (FFPE) samples. FFPE samples then underwent dewaxing and antigen retrieval prior to staining.

### Antigen retrieval of formalin fixed paraffin embedded samples and fixation of frozen samples

For removal of paraffin slides were immersed in xylene (three times for 5 min), rehydrated with graded ethanol (twice for 5 min in 100%, once for 5 min in 90%, once for 3 mins in 70%, once for 3 mins in 50%, once for 3 mins in 30%) and transferred to deionised water for 5 mins twice. Sections were then transferred to a citrate buffer (PickCell Buffer Type A, PickCell Laboratories, Leiden, the Netherlands) and underwent antigen retrieval in a model 2100 steam antigen retrieval unit (PickCell Laboratories, Leiden, the Netherlands) for 20 mins, followed by a 2 hour cool down. Sections were then washed five times in deionised water followed by two ten minute washes in Tris Buffered Saline. Frozen sections were thawed and fixed in ice cold acetone for five minutes followed by one wash with Tris Buffer Saline.

### Immuno-staining

Sections were blocked with 0.25% casein for 10 minutes at room temperature, then incubated with the primary antibodies listed in Supplementary Table 2 for one hour at room temperature. The primary antibodies were detected with the corresponding isotype-specific goat anti-mouse or goat anti-rabbit secondary antibodies conjugated to a fluorochrome (488, 555 or 647; Invitrogen, CA, USA). DAPI was included at 0.0005% w/v with secondary antibody. Following staining, background autofluorescence was quenched by bathing slides in 0.1% Sudan Black B Solution for 15 minutes followed by two brief washes in TBS then three 1 hour washes in TBS. All data shown of liver tissue are representative of at least 3 or more biological replicates and were consistent findings among all livers assessed. The number of bioligical replicates in which each antigen was assessed in liver tissue is presented in Supplementary Table 3. Data of positive control tissue presented in the supplementary information was performed in one biological replicate.

The slides were mounted using Prolong Gold (Invitrogen). Sections were visualized with a Nikon Ni-U fluorescent microscope equipped with the epi-fluorescent filters: UV, 450–490 nm, 530–560 nm and 590–650 nm. Images were acquired at room temperature using 4x/0.13 numerical aperture (NA), 10x/0.45 NA, 20x/0.75 NA and 40x/0.95 NA Nikon objectives, a SPOT Pursuit 1.4MP camera and Spot v.5.0 Spot software (Sterling Heights, USA). Images were generated using Cytosketch (CytoCode, Auckland, New Zealand) and figures were formated using Adobe Illustrator (Adobe Systems, Mountain View, CA).

### Quantative studies and statstical analysis

Quantifying differences in cell number and marker expression is a highly complex process using immunofluorescence^18^,^19^, however, in order to provide quantified evidence of observed differences in the distribution of fluorescence of LSEC markers (CD36, CD32, CD14 and LYVE-1) between Z1 and Z3, ImageJ (National Institutes of Health, Bethesda, Maryland, USA) was used to assess the mean intensity of fluorescence in a greyscale image in 5 randomly chosen Z1 areas and 5 randomly chosen Z3 areas of the same size in images from 3 different patients. For each liver, the mean intensity of fluorescence was assessed in different zones of the same image (to ensure the same conditions of immunostaining, visualization and image capture).

To quantify intensity of expression for PT and CV makers ImageJ was again used to assess the the highest level (or “Max”) intensity of fluorescence in 5 different PTs and 5 different CVs within the same image(again to enure the same conditions of immunostaining, visualization and image capture). This was repeated for 3 livers.

A linear mixed model (LMM), accounting for multiple measurements made on each liver, was fitted to the log-transformed intensity data for each marker (CD14, CD36, CD32, LYVE-1 in the case of LSEC markers and CD146, aSMA, and laminin in the case of PT and CV markers). The log-transformation was required to meet the underlying assumption of homogeneity of variance of the LMM. The results from the pairwise comparisons of means between zones (or PT and CV) were back-transformed to their original scales and are reported as ratios, this being the median intensity of Z3(or PT) relative to the median intensity of Z1(or CV). Also reported are 95% confidence intervals for these ratios and p-values resulting from the t-tests.

The results of the quantative and statistical analysis are reported in the Supplementary Information (see Supplementary Table 4 and Supplementary Table 5).

## Results

**Figure 1:**
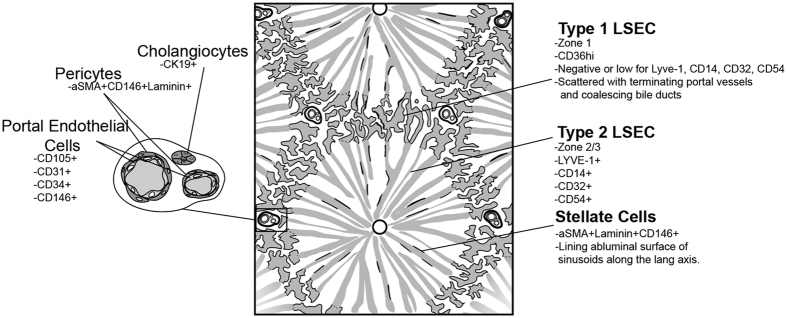
Graphical representation of the liver lobule highlighting the findings of this study. Microscopically the liver can be described by its functional unit, the acinus. Blood drains from the portal vein and hepatic artery and mixes in the sinusoids and eventually drains to the CV. Z1 refers to area between two PT, and hepatocyte function changes as blood drains towards the CV. Hepatocytes are lined by two distinct subsets of LSEC, Type 1 which are located in Z1, and Type 2 which are located in Z2 and Z3. Bile is produced by hepatocytes and flows back via bile caniculi (Canals of Hering) towards the PT and eventually enters the bile duct. The authors acknowledge the assistance of Vivian L. Ward in the production of this figure.

Figure 1 presents an illustrated summary of the findings in this manuscript.

### Zone 1 contains terminating portal capillaries, coalescing bile ducts and LSEC that have a distinct phenotype from LSEC in zones two and three.

**Figure 2:**
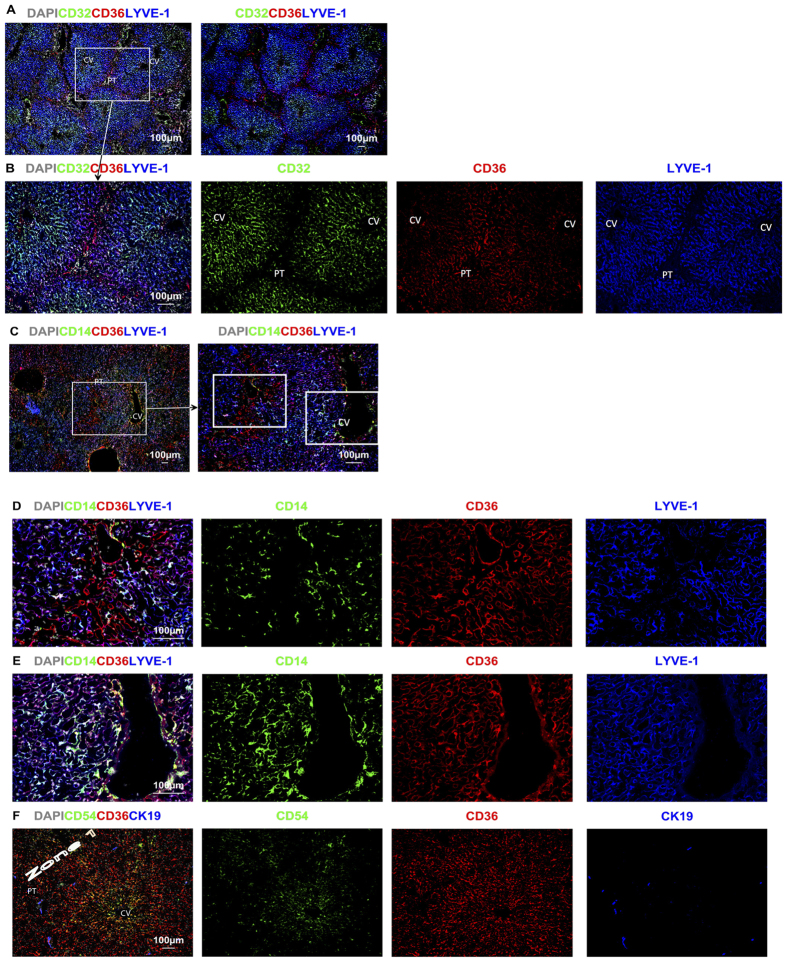
CD36 and LYVE-1 identify distinct subsets of LSEC. (**A**) CD36^hi^LYVE-1^lo^CD32^−^ Type 1 LSEC are found in Z1. LYVE-1^hi^ Type 2 LSEC line hepatocytes in Z2 and Z3 of the acinus (**A–D**) Z2 and Z3. CD32 (**A**) is most prominent closest to the CV on Type 2 LSEC. Type 2 LSEC also express CD14 (**C–E**). Z1 is clearly seen as being negative or low for CD32, LYVE-1 and CD14 and bright for CD36. (**F**) CD54 is also expressed by Type 2 LSEC, CK19 is used to identify PT and bile ducts in Z1.

**Figure 3:**
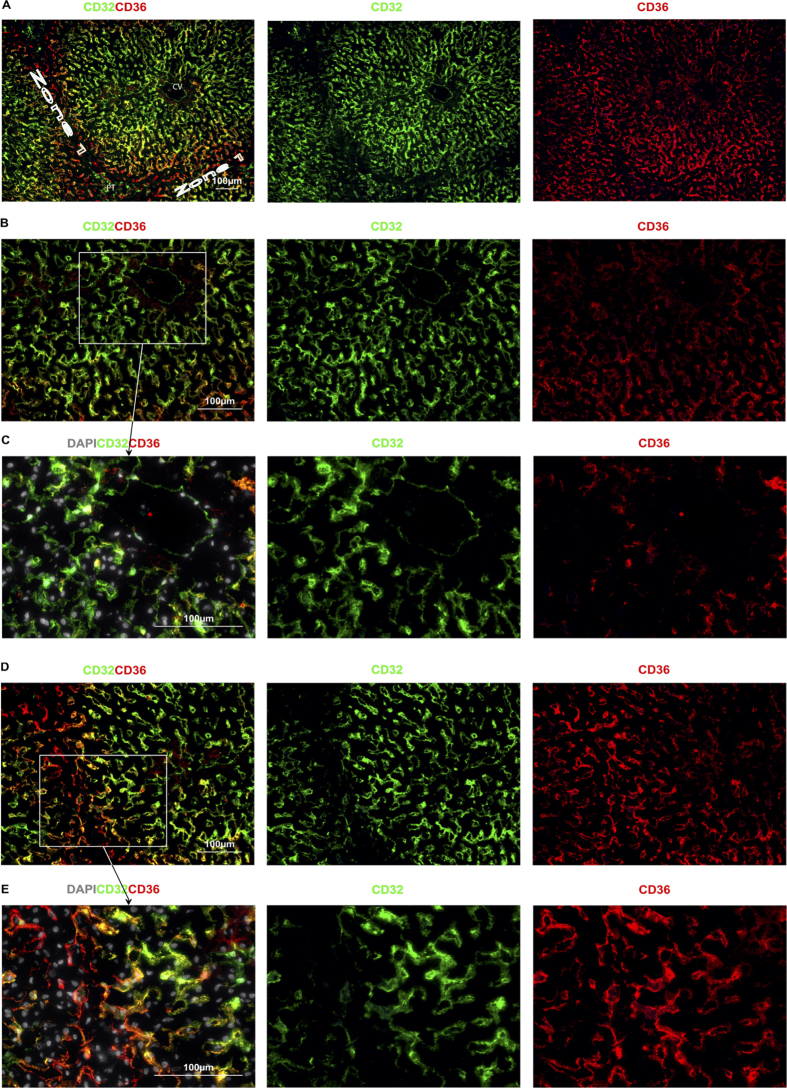
CD32 and CD36. Representative Immunofluorescent Microscopy Images showing CD32 and CD36. (**A**) CD36 is expressed by LSEC in all zones of the lobule but is brightest in Z1 (Type 1 LSEC). Much like LYVE-1, CD32 is expressed brightly on LSEC throughout the lobule except on CD36^hi^ Type 1 LSEC. (**B** and **C**) CD32 is brightest in Z2 and near the CV and although present, CD36 is lower on LSEC near the CV. (**D** and **E**) a transition zone of orange cells is visible on the border of Z1 as cells that brightly co-express CD32 and CD36. Type 1 LSEC are orientated in a more web-like pattern than Type 2 LSEC.

**Figure 4:**
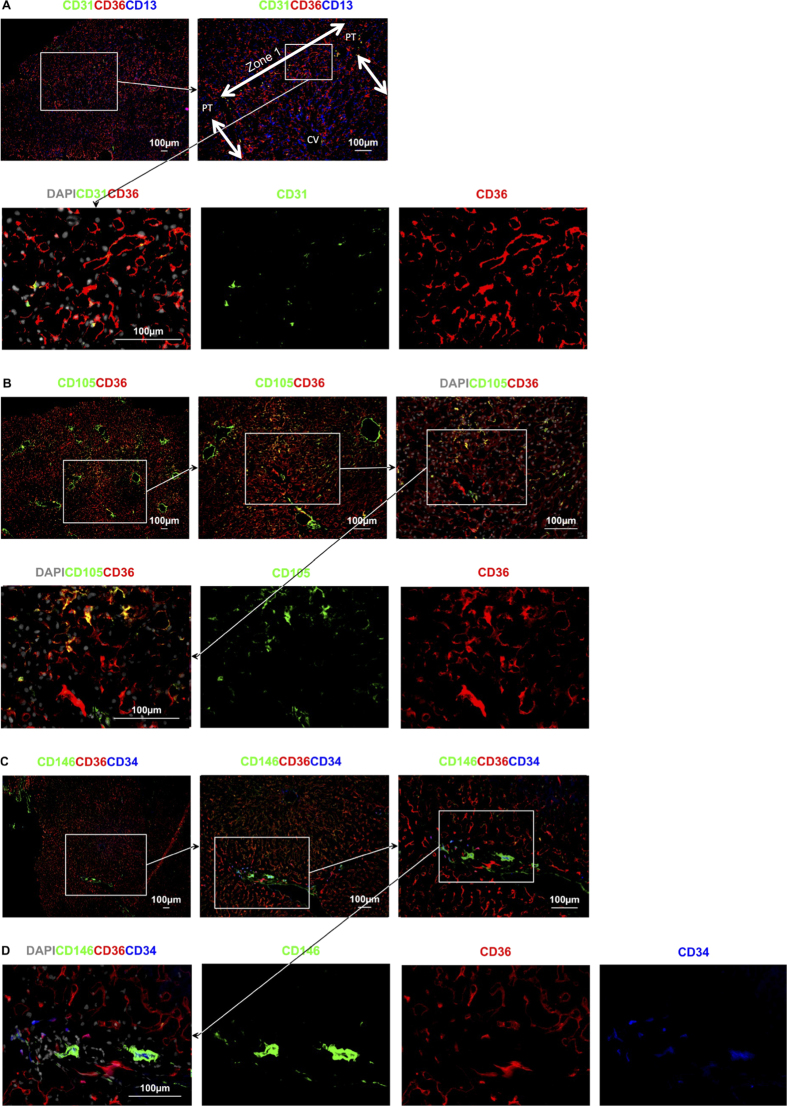
Z1 contains Type 1 CD36^hi^ LSEC and terminating PT vasculature. **(A** to **D**) Z1 in **A** is indicated by double pointed arrows, the inset is a higher magnification image. Terminal branches of the portal vein and hepatic artery, as shown by CD31 (green in **A**), CD34 (blue in **C** and **D**), CD105 (green in **B**) and associated CD146^+^ pericytes are replaced by sinusoidal endothelium. (**C** and **D**) CD146 can be seen very brightly in the Z1 region closest to the PT, presumably on or wrapped around terminal branches of arterial vascuature as it leaves the PT.

Two different subgroups of LSEC are identifiable by their levels of expression of the cell surface markers CD36 and LYVE-1 as shown in Fig. 2. Type 1 CD36^hi^ LSEC occupy Z1 of the acinus, delineated in Fig. 2F by coalescing bile ducts expressing CK19 in the PT and the area between adjacent PT. Type 1 CD36^hi^ LSEC express at most very low levels of LYVE-1 (Fig. 2). These Type 1 CD36^hi^ LSEC also have at most low expression of CD32 (Fig. 3). In contrast Type 2 LSEC in Zone 2 and 3 (Z2 and Z3) have high expression of LYVE-1 (Fig. 2) and CD32 (Fig. 3). Expression of CD36 in these Type 2 LSEC varies from moderate in Z2 to low in Z3 (Figs 2A,D, 3D and E) but is almost always lower than for Type 1 LSEC. These LYVE-1^hi^CD32^hi^CD36^mid-lo^ Type 2 LSEC also express high levels of CD14, and CD54 (Fig. 2C–F) while Type 1 CD36^hi^LYVE-1^lo^CD32^lo^ LSEC do not express these markers (Fig. 2C–F). Neither of the two LSEC subpopulations express the classical endothelial markers CD31 or CD34 (Fig. 4) which are expressed on both PT and CV vessels.

The patterns of distribution of Type 1 and Type 2 LSEC were crudely quantified using ImageJ. These data are presented in the Supplementary information and show that Z3 LSEC are brighter in expression of CD14, CD32, and LYVE-1, with Z1 LSEC being brighter for CD36 as was expected, all p-values are less than 0.05.

The Type 1 CD36^hi^ LSEC have a web-like distribution radiating out from the PT. Sinuses in Z1 are the first to receive blood from the portal circulation, and consistent with this, Type 1 LSEC are interspersed with terminating branches of vessels from the PT that can be labelled using CD31, CD34, or CD105 (Fig. 4A–D). Although Type 1 LSEC do not express CD31 and CD34, at least some do express CD105 (Fig. 4B), suggesting continuity between the terminating portal capillaries and Type 1 LSEC. However terminating portal vessels in Z1 of the lobule are surrounded by CD146^hi^ pericytic cells, while they do not surround Type 1 LSEC (Fig. 4C–D).

**Figure 5:**
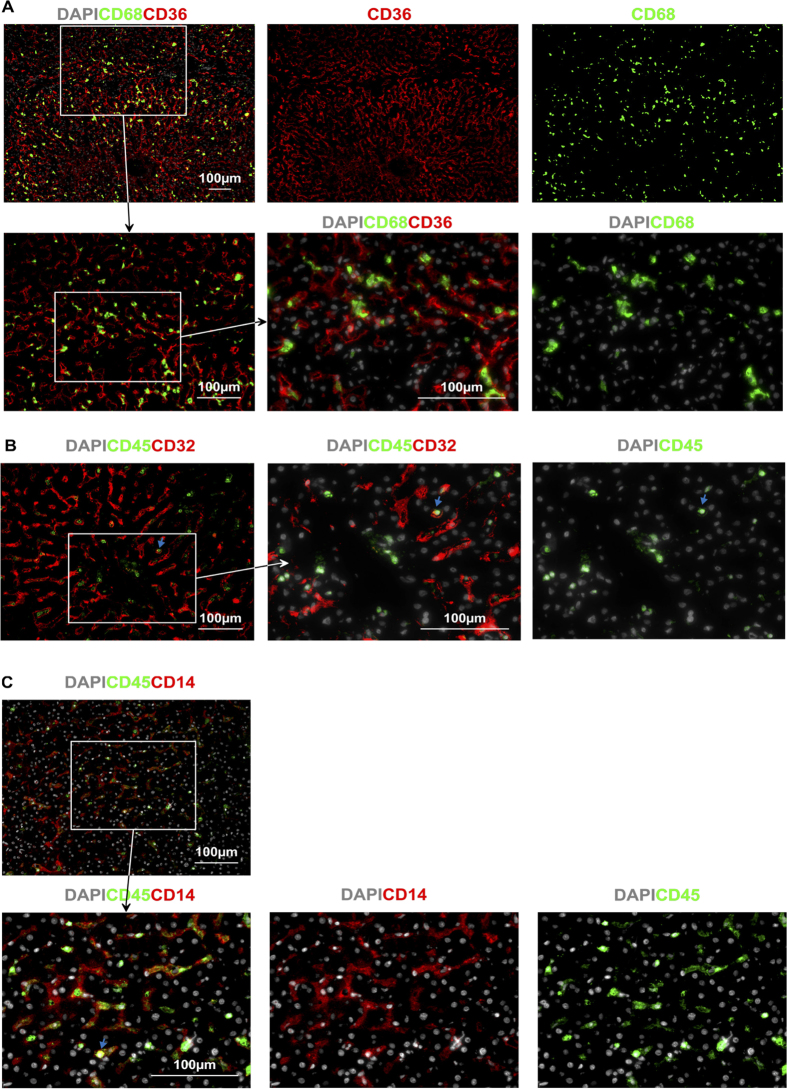
LSEC can be distinguished from CD36^+^, CD14^+^ and CD32^+^ APC. **(A**) CD36^hi^ Type 1 LSEC are distinct from CD68^+^ KC that can be seen occupying the sinusoidal lumen. This is exemplified by the lack of CD68 and CD36 co-expression. (**B**) Type 2 LSEC (in this slide represented by CD32) are spindle shaped and CD45^−^, as opposed to what appears to be a transiting monocyte identified by the arrow, Type 2 LSEC can also be identified by CD14 and are distinct from KC (as seen by the spindle shaped CD45^−^. Type 2 LSEC are also distinguishable from transiting monocytes (the round CD14^hi^CD45^hi^ cells as identified by the arrow in this example) by using CD45.

LSEC could be distinguished from Kupffer cells (KC) that are found in close proximity but are distinct cells that express CD68 (see Fig. 5A). Type 2 LSEC that are CD32^hi^ (as described above) are also found to be CD45^−^ (see Fig. 5B) and sinusoidal CD45^+^CD14^+^ cells can clearly be differentiated from CD45^−^CD14^+^ LSEC (Fig. 5C). Fig. 5A and 5C also highlight the close proximity of LSEC and macrophages on the luminal side of the vessel. Hence the dominant signal for CD32 in Fig. 3 derives from Type 2 LSEC rather than from KC or sinusoidal monocytes (Fig. 5B).

### Distinctive molecular phenotypes in the portal tract and the central vein

**Figure 6:**
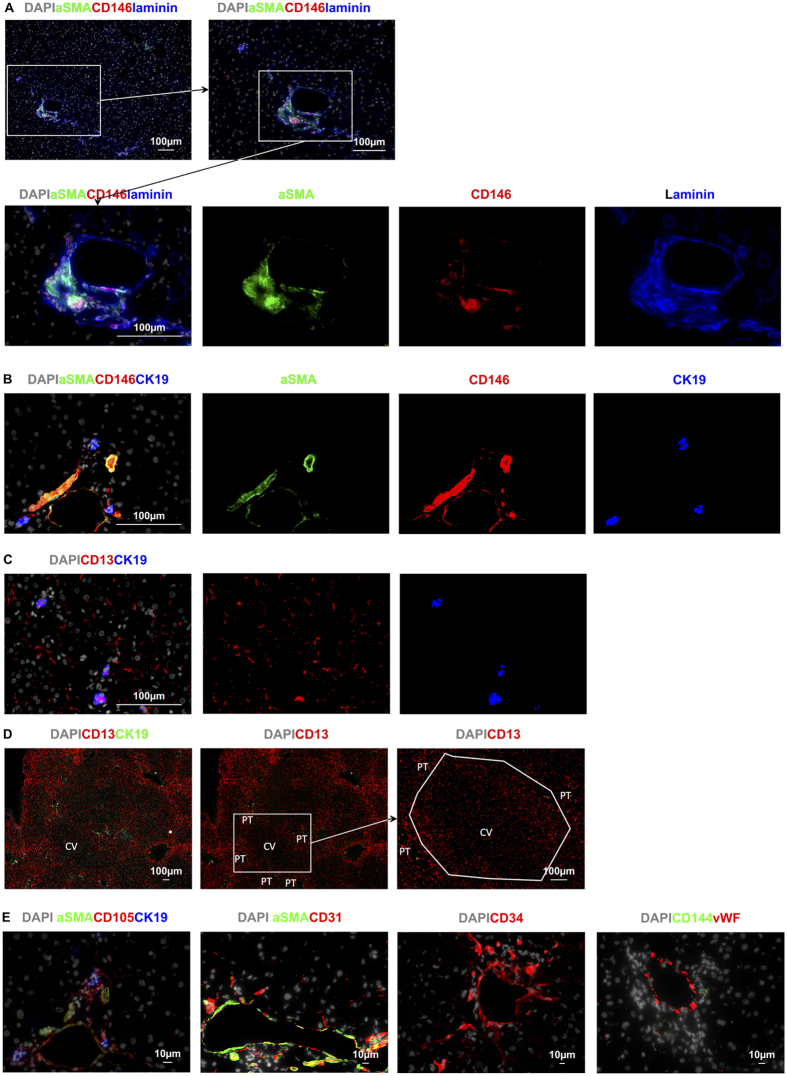
Identifying portal tracts using specific markers for pericytes. **(A**) aSMA, laminin and CD146 is expressed by pericytes surrounding the hepatic artery and portal vein. These markers can be used individually or in combination to identify portal tracts. (**B**) Cytokeratin 19 (CK19) is brightly expressed on bile ducts, aSMA and CD146 identify pericytes surrounding portal vasculature. (**C**) CD13 (red) identifies bile caniculi coursing towards bile ducts, the luminal surface of which are also CD13^+^. PTs can be seen surrounding central veins. As bile ducts coalesce their CD13 appears more prominent, the white outline in third image in **D** roughly highlights the areas of Z1 between PTs. (**E**) Endothelial cells in the PT were positive for CD146, CD105, CD31, CD34, and von Willebrand factor. CD144 was expressed in the PT.

**Figure 7:**
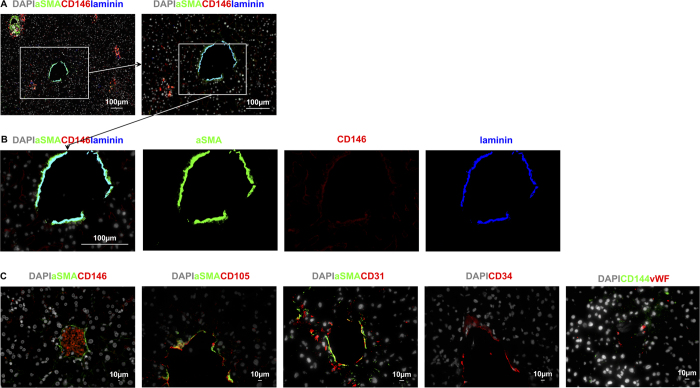
Identifying systemic vascular endothelium on the CV. **(A** and **B**) aSMA and laminin are brightly expressed on cells around the outside of the CV and CD146 is present on CV endothelia but is much weaker than on PT vessels. (**C**) CV endothelia express CD146, CD105, CD31, CD34, and von Willebrand factor. CD144 was present in the CV. This particular image highlights the autofluoresence of red blood cells in a central vein.

Vascular pericytes expressed CD146 (Fig. 4C,D) as well as aSMA and laminin (Figs 6 and 7). Pericytes surrounding the portal vein and hepatic artery had exceptionally bright co-expression of aSMA, CD146, and laminin (Fig. 6A). In the CV, while aSMA and laminin were also very brightly expressed by pericytes, CD146 expression was notably duller than on the pericytes surrounding PT vessels (Fig. 7A,B). The central vein also had less marked clustering of nuclei (Fig. 7). Staining for these markers therefore provided a convenient method to definitively identify PT and CV scattered amongst sinusoids and provided a framework for establishing the acinar distribution of the expression of markers by LSEC.

The vascular endothelial cells within the portal vein and hepatic artery showed subtle differences from those in the central vein. Endothelial cells in all the vessels expressed the systemic endothelial markers CD105, CD31, and CD34, but not aSMA (Figs 6E and 7C). Endothelial cells in the PT also expressed CD146 (Fig. 6A,B) while those in the CV had a much weaker staining of CD146 (Fig. 7A–C). Hence CD146 expression was much weaker overall, in both the endothelia and the pericytes, in the CV than in the vessels of the PT. A similar pattern was also seen for vWF(von Willebrand factor) which was very brightly expressed on the luminal surface of PT vessels (see Fig. 6E, Supplementary Figure 2), and less brightly expressed but also prominent on the CV luminal surface (see Fig. 7C, Supplementary Figure 2). CD144 staining appeared to be present on PT, CV and cells in Z1, expression appeared to be relatively weak when compared to positive control tissue (See Figs 6E and 7 C, and Supplementary Figures 2 and 3), lobular CD144 was weak and inconsistent.

The relative brightness of pericyte markers between PT and CV were also crudely assessed in a quantative manner, the difference in the mean of highest fluoresence of markers quantitatively assessed (aSMA, CD146 and laminin) comparing PT and CV all had a p-value of <0.05 (these data are presented in Table 5 of the Supplementary Information).

CD36 expression by vascular endothelial cells was variable but more prominent on PT than CV vessels (see Figs 2D,E, 4C and D). As mentioned above, vascular endothelial cells of the PT and CV can be distinguished from LSEC using systemic vascular markers such as CD31 or CD34, and are associated with pericytes that brightly express laminin, aSMA and CD146 (see Figs 6A,B, 7A and 7B). Vascular endothelia can also be identified by their location (within PT or in the centre of the lobule) and their morphology, as they tend to have a characteristic round shape and wider lumen than LSEC.

**Figure 8:**
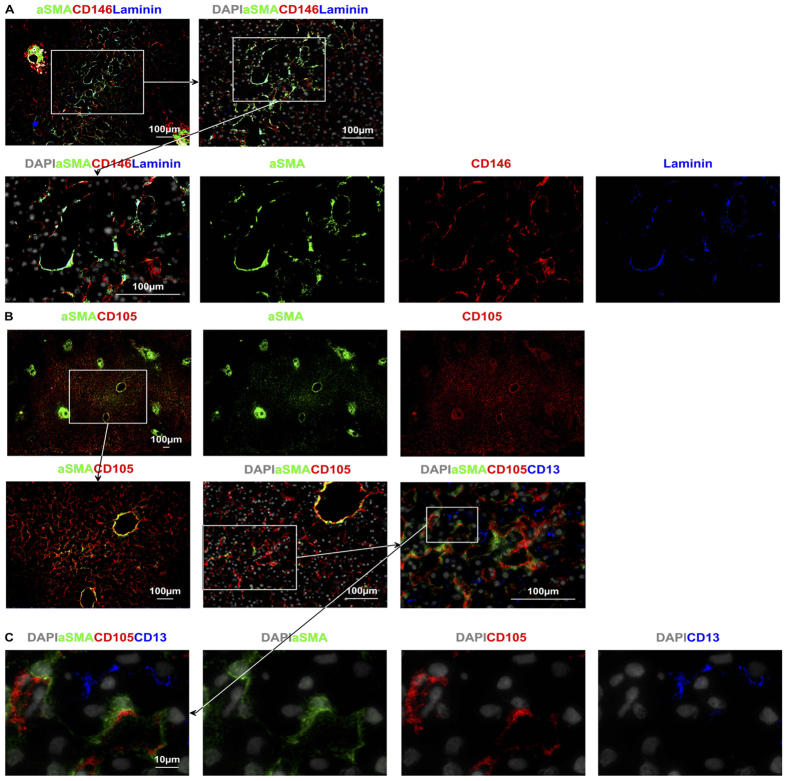
Immunofluoresence microscopy allows for an appreciation of the complexity of the perisinusoidal space. **(A** and **B**) aSMA and laminin are expressed by cells in the “long axis” between two CV and are closely associated with LSEC. These cells have a higher expression of CD146 and may represent lobular pericytes, or stellate cells. LSEC in this region also express CD105. The expression of aSMA of these cells indicates a potential role in modulating blood flow within the lobule. (**C)** Higher magnification images highlight the complexity of the perisinusoidal space, CD105 on red identifies LSEC, aSMA stellate cells lie between hepatocytes in the Space of Disse, and CD13 identifying Canals of Hering between abutting hepatocytes.

Within the PT, biliary epithelial cells could readily be distinguished by their bright expression of cytokeratin 19 (CK19) and their co-expression of CD13 (Fig. 6C–D). The entire biliary tree could also be identified using CD13 which was expressed on the luminal surface of the biliary epithelium, from the Canals of Hering coursing between hepatocytes in the liver lobule, to the inner surface of the bile ducts in the PTs (Fig. 6C). The Canals of Hering appear to increase in size (Figs 4A and 6C) as they coalesce, heading towards the CK19^+^ biliary epithelia in Z1 which eventually drain into bile ducts within the PT. Although it is recognized that CD13 is also expressed by myeloid cells^20^ the CD13 present on the Canals of Hering is located between hepatocytes (Figs 4A and 8C) and follows a distribution of distinctive linear (canal) staining devoid of CD45 and CD14 and without associated nuclei.

### aSMA^+^ stellate cells are predominantly located in areas distal from the PT

Stellate cells have been reported as positive for alpha smooth muscle actin (aSMA) and laminin. IFM for these markers identified a population of aSMA^+^ laminin^+^ perisinusoidal cells, as well as pericytes in the vessel walls of the PT and CV (described above). aSMA^+^ laminin^+^ cells not associated with portal or central vein blood vessels were positioned on the abluminal (parenchymal) surface of the sinusoids, in the space of Disse between LSEC and hepatocytes and are distinct from LSEC (Fig. 8C and D). They occupied a distinctive location in the area of the liver lobule that runs between adjacent central veins (across all three zones of the acinus – often referred to as the long axis) but were absent from regions of the lobule close to the portal tract (Fig. 8A and B). The interface between the sinusoidal lumen and the Space of Disse is complex (Fig. 8C) and is composed of a heterogeneous collection of cells that have a similar gross structure and are located in a very close proximity to each other. Stellate cells, KC, and LSEC all have a spindle shape and are indistinguishable without molecular markers.

## Discussion

An improved understanding of the endothelial and epithelial structures within the human liver is likely to provide insights into liver-specific disease processes and identify new targets to monitor and combat liver disease. In this paper we demonstrate heterogeneity of LSEC in normal human liver, identifying two LSEC types that are distinctly distributed in the liver acinus. We also characterize the molecular phenotype and distribution of portal structures including blood vessels and pericytes, and the distribution of stellate cells in relation to different liver endothelia.

CD36, LYVE-1 and CD32 have been used previously to identify LSEC in single colour stains 12,14,15,16. Our data demonstrate co-staining of two distinctive populations of LSECs, CD36^hi^LYVE-1 ^lo^CD32^lo^ and LYVE-1^hi^CD32^hi^CD36^mid-lo^ that map to distinctive acinar zones of the liver lobule. We therefore propose these distinctive populations be designated as Type 1 and Type 2 LSEC.

Previously, it had been noted that LYVE-1, although specific for LSECs in the liver^14^,^15^, stain LSEC predominantly in Z2^14^. Here we demonstrate that by introducing a CD36 co-stain along with LYVE-1, LSECs in all acinar zones can be visualised. We also show that CD32 is co-expressed by Type 2 LYVE-1^+^ LSEC, but not by LYVE-1^-^ Type 1 LSEC, and although it may also stain KCs or sinusoidal monocytes, CD32 offers an alternative combination with CD36 to delineate the two subsets of LSECs. Hence while our data on LYVE-1 are largely consistent with previous findings^14^, co-staining LSEC with other markers reveals the full extent of LYVE-1 staining across Z2 and Z3, and the lack of LYVE-1 in Z1.

The different zones of the acinar model of the liver lobule have been classically defined by their perfusion from afferent PT vessels; blood with a relatively high oxygen content that has been supplied by the hepatic artery and portal vein enters the lobule in Z1 through infiltrating capillaries from PT vessels, and moves down a pressure gradient to eventually exit Z3 into the CV^21^. This zonal distribution is reflected in the function of hepatocytes^22^ and LSEC phenotype is affected by adjacent hepatocytes^6^,^7^. Hence, blood flow into zones and across endothelial cells may relate to zone-specific functions (such as the entrance of leukocytes into the tissue^23^,^24^, or egress from the tissue)^25^. Our phenotypic description of endothelial cells in different zones that follow an acinar distribution provides further evidence for the zones of the liver acinus representing the functional microanatomy of the liver lobule.

A further investigation of the microanatomy of the liver lobule using vascular reconstruction in mice by Matsumoto and Kawakami^23^ revealed a more accurate vascular distribution of zones that closely represents the staining patterns of Type 1 and Type 2 LSEC (see Figs 2 and 3). Areas of high oxygenation and perfusion are not strictely restricted to areas between PTs but also extend into the area immediately surrounding the PT in a crescent shape as terminating capillaries from Z1 areas extend out in three directions from each PT (each of these extensions extends to another PT and creates the characteristic hexagonal pattern seen in the lobule). Our data supports this pattern of staining and is presumed to be related to the relative composition and hydostatic pressure of blood in the different zones of the lobule.

Type 1 LSEC are distributed in a web-like pattern and are restricted to Z1. Type 1 LSEC directly drain blood from terminating portal vessels into sinuses with a wider luminal space than Type 2 LSEC. This increased luminal diameter may facilitate the reduction in the velocity of blood flow into the lobule^26^ and the lack of adhesion receptors on Type 1 LSEC (e.g. CD54) may be indicative of this region as an entry point into the lobular sinuses. Type 1 LSEC express high levels of CD36 and very little of the other LSEC markers previously identified (e.g. LYVE-1, CD32, CD14). As a result, despite being expressed by other cells such as APC and vascular endothelial cells^15^, we identify CD36 as the most universal LSEC marker of those described to date. The role of CD36 on LSEC is presumed to relate to their scavenging function^15^, and the higher CD36 expression by LSEC in Z1 that we describe implies their scavenging role is focused on a quite different set of ligands than the LSEC in Z2/3.

Type 2 LSEC are distributed in a concentric pattern in Z2 and Z3. They brightly express LYVE-1, a marker that is involved in adhesion and cell migration^6^, and CD32, the low affinity receptor that binds the Fc portion of IgG. Type 2 LSEC also express immune receptors such as CD14, the co-receptor for lipopolysaccharide (LPS), and the adhesion receptor CD54. Hence in contrast to Type 1 LSEC, Type 2 LSEC have a molecular phenotype consistent with a role in adhesion of blood derived leukocytes and clearance of blood-derived antigen in the lobule that is entering the liver from the systemic circulation and the gut. The exact mechanism by which cells enter the Space of Disse is unclear^15^ but LYVE-1 is a hyaluronic acid (HA) binding protein and HA has been implicated in the extravasation of cells across the liver sinusoid^15^. It would be fascinating to assess the expression of other markers involved in cell arrest and migration such as Vascular Adhesion Protein 1, and stabilin 1, with the Type 1 and Type 2 LSEC model as a framework. Ultimately, these data provide further evidence for a functional distribution and microenvironment within the liver lobule.

Zonal distributions of endothelial cell surface marker patterns within the lobule^16^ have previously been described using light microscopy and identified terminating capillaries from vessels of the portal tract draining into branching patterns of what appeared to be LSEC between Z1 and Z2/Z3. The marker IF10 was found predominantly in Z1 while CD16 was predominantly found outside Z1, putatively on LSEC^16^. IF10 labels continuous endothelia as opposed to sinusoidal endothelium (which lacks a basement membrane and is not continuous) and our data suggests it is likely to have identified terminating portal vessels in Z1. CD16 is brightly expressed by KCs that are located in Z2 and Z3 and have a spindle shape resembling LSEC^27^, so co-staining will be advisable to confirm CD16 expression by LSEC. However given the expression of CD32 by Type 2 LSEC in our work, it seems likely that Type 2 LSEC may also express CD16, another Fc receptor.

In our hands, LSEC do not consistently express systemic vascular markers such as vWF, CD144, CD34 and CD31. This lack of classic endothelial markers on LSEC must be taken into consideration when looking at a single cell suspension of liver. Numerous studies have used systemic markers such as CD31 to sort endothelia from liver immunomagentically^5^,^28^ or using Fluorescence Assisted Cell Sorting (FACS)^6^ but our data suggest CD31 is likely to select endothelial cells of the CV or PT. In order to accurately identify LSEC, and especially their two distinctive subsets, a combination of markers such as LYVE-1, CD32, CD36, CD14, and CD54 will be required.

PTs are highly cellular and contain different types of systemic vascular endothelial cells that are distinct from those found in the lobule. Vascular endothelia of the hepatic artery and portal vein express markers of systemic endothelial cells such as CD31, CD34, CD105, and CD146^29^. These systemic vessels are surrounded by aSMA^+^laminin^+^CD146^+^ cells that are likely to be pericytes^30^ and infiltrate into Z1 from the PT where they merge with the network of CD36^hi^ LSEC. The distribution of PT endothelia terminating in Z1 is supported by Scoazec *et al*.^16^ who described the presence of systemic endothelial cell markers that radiated into Z1 from the PT.

Beyond draining blood into the sinusoids it has been postulated by Guow *et al*. that the distribution of CD34 and bile ducts represents the vasculature support for these bile ducts that are also found in Z1^31^. Bile ducts are formed from coalescing Canals of Hering that drain bile towards the PT. The Canals of Hering brightly express the aminopeptidase CD13 on their luminal surface that can be traced from Canals of Hering in Z3 to the luminal surface of CK19^+^ bile ducts in Z1 and the PT. These findings support the notion that blood enters the lobule via terminating systemic vessels prior to entering CD36^hi^ sinusoids, and bile flows in the opposite direction towards Z1 before entering CK19^+^ bile ducts which then drain into the portal tract.

We provided quantified evidence of the existence of Type 1 and Type 2 LSEC, and the differences in intensity of markers expressed in PT and CV(see Supplementary Information), but quantification of cells such as sinusoidal endothelia must be interpretted with caution, our quantification provides further evidence of our observed findings, but it must be emphasized that the findings in this paper are intended to be descriptive.

Stellate cells have been described as being aSMA^mid^^32^, CD271^+^ 33, and are CD146^+^ in animals^34^, lining the abluminal surface^32^ of the sinus. We used aSMA and CD146 to clearly demarcate stellate cells from adjacent CD105^+^ LSEC and describe a lobular distribution of these cells. Our findings indicate that aSMA^+^ stellate cells are distributed at a distance from PT, along the axis running between CVs, in the area of the lowest hydrostatic pressure for each zone of the acinus^35^. Interestingly these cells are not present in peri-portal regions and their aSMA expression may indicate a contractile function that provides a means of promoting blood flow towards central veins.

Dysregulated contractility of aSMA^+^ stellate cells my play a role on both vascular congestion and increased vascular resistance through their effect on luminal diameter^36^. Further to that, stellate cells also contribute to portal hypertension as a result of extracellular deposition of collagen in cirrhosis as the major collagen depositing cells in the liver lobule^37^. Their role as collagen depositing cells and their location in the lobule provides some insight into the distribution of collagen deposition seen in perivenular cirrhosis^38^. Our data define the distribution of stellate cells in the lobule and provide further evidence for their role as sinusoidal pericytes.

An understanding of the molecular phenotype of liver vascular structures, epithelial and stromal cells is vital in identifying any pathological changes and potential therapeutic targets. Immune cell infiltrates are present within the liver lobule in many disease states^39^. Having knowledge of the phenotype of LSEC provides an anatomical framework to investigate the mechanisms employed by leukocytes to enter the parenchyma and PT extravascular space^28^.

LSEC may play a pivotal role in tolerization via antigen presentation to CD8^+^ T cells^40^ and capillarization of LSEC in animal models of cirrhotic liver disease contributes to immune dysfunction^41^. Characterising which subset of LSEC predominantly perform this function will be useful in investigating these cells as potential targets for future therapies. The differential expression of adhesion molecules we detected provides a framework to further characterize the interactions of these cells with immune cells.

Other non-parenchymal cells in the liver such as KCs have yet to be thoroughly characterized, but are also clearly heterogeneous^42^. Whether or not this heterogeneity follows an acinar distribution will provide insights into the roles these cells play in health and disease. By defining the acinar distribution of LSEC sub-populations with which cells like KCs interact, this work provides a framework for the study of other liver non-parenchymal cells in both health and disease.

In conclusion, we have defined two distinct populations of LSEC, as well as distinctive markers of the systemic vascular endothelium and their network of supporting cells. Our findings will enable further exploration of the phenotype and function of endothelial cells in normal liver, and more accurate assessment of how they change in disease, and how they might be targeted in therapy.

## Additional Information

**How to cite this article**: Strauss, O. *et al*. Immunofluorescence identifies distinct subsets of endothelial cells in the human liver. *Sci. Rep.* **7**, 44356; doi: 10.1038/srep44356 (2017).

**Publisher's note:** Springer Nature remains neutral with regard to jurisdictional claims in published maps and institutional affiliations.

## Supplementary Material

Supplementary Information
