# Supplementary material for: Immunofluorescence identifies distinct subsets of endothelial cells in the human liver
Source: Sci Rep. 2017 Mar 13;7:44356. doi: 10.1038/srep44356 (PMC5347010; doi:10.1038/srep44356)
Supplement: Supplementary Information [file srep44356-s1.pdf]

Supplementary Material

Title: Immunofluorescence identifies distinct subsets of endothelial cells in the human liver.

Short title: The Human Liver Endothelial cells.

Table of Contents

Supplementary Figure 1 4

Supplementary Figure 2 6

Supplementary Figure 3 8

Supplementary Figure 4 11

Supplementary Tables 12

Table 1 Patient details 12

Table 2 Antibodies used 13

Table 3 Number of biological replicates performed for each antigen presented. 14

Table 4 - Findings comparing fluorescence in LSEC makers between zones. 15

Table 5 - Findings comparing intensity of fluorescence in PT and CV makers. 15

List of investigators

1st and corresponding Author.

Otto Strauss, MBChB, PhD(1,2,3).

Department of Surgery, Faculty of Medical Health Sciences, University of Auckland, Level 12 Support Building, Auckland Public Hospital, Park Road, Auckland.

Contribution: Designed experiments, acquired and analysed data, wrote

manuscript.

2nd Author

Anthony Phillips, MBChB, PhD(1,2,3).

Contribution: Provided intellectual input regarding manuscript structure and study design. Reviewed manuscript. Supervised work.

3rd Author

Katya Ruggiero, PhD (4).

Contribution: Performed all the statistical analysis presenting in the supplementary information.

4th Author

Adam Bartlett, MBChB, PhD(1,2,5).

Contribution: Provided intellectual input regarding manuscript structure and study design. Reviewed manuscript. Supervised work.

5th Author

P. Rod Dunbar, MBChB, PhD(2,3,5).

Contribution: Provided intellectual input regarding manuscript structure and study design. Reviewed manuscript. Supervised work.

[1]Department of Surgery, Faculty of Medical Health Sciences, University of

Auckland, Auckland, New Zealand.

[2]Maurice Wilkins Centre, University of Auckland, Auckland, New Zealand

[3] School of Biological Sciences, Faculty of Science, University of Auckland, Auckland, New Zealand

[4] Department of Statistics, University of Auckland, Auckland, New Zealand

[5] These authors contributed equally.

Supplementary Figure 1

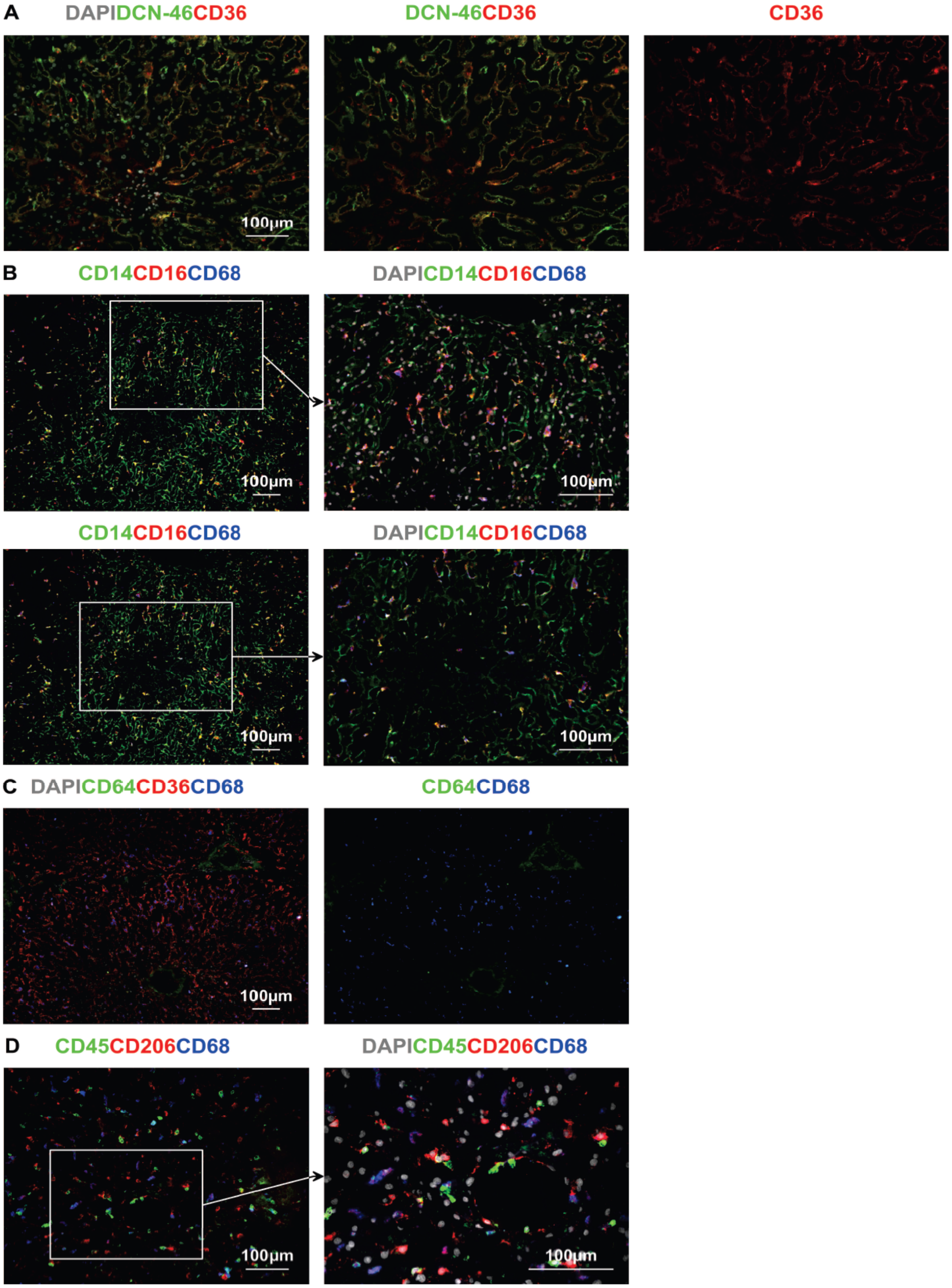

Supplementary Fig. 1

(A) DCN-46, a marker for CD299 is expressed by LSEC in Z2 and Z-3 as

identified by CD36 staining. (B) When assessed with other FcReceptors besides CD32, LSEC are predominantly negative for CD16. CD16 is highly expressed by $CD68^{+}$ KC.(C) LSEC are also negative for CD64. (D) CD206 is expressed by both $CD68^{+}$ KC and $CD45^{-}CD68^{-}$ cells that are likely to be LSEC. This expression does not appear to be present in Z1 which would be in keeping with Type 2 LSEC.

Supplementary Figure 2

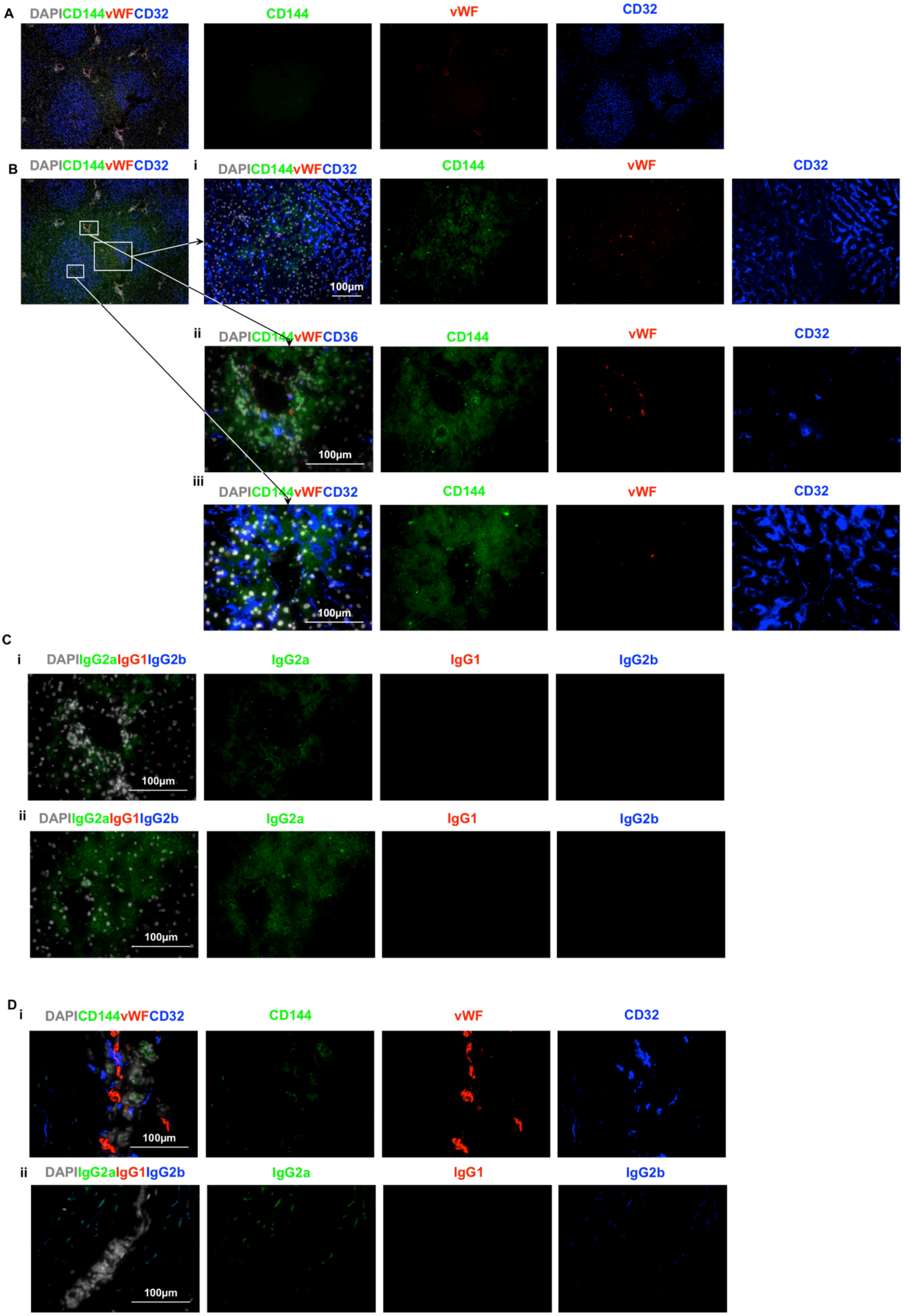

Supplementary Fig. 2

This image highlights that von Willebrand Factor (vWF) is brightly expressed on the luminal surface of PT vessels and also present in the luminal surface of CV (see B - ii, B - iii). CD144 was dimly expressed by cells in the PT, this was more marked on smaller structures that have a morphology consistent with hepatic arteries (B - ii). This pattern of staining is distinct from the non-specific pattern of autofluorescence seen in the negative control samples of PT(C - i) and CV(C - ii) which did not undergo incubation with primary antibodies. Skin biopsy tissue was used as a positive control for both vWF and CD144 as both are expressed by vascular endothelial cells in the dermis (D - i), this staining pattern is distinct from the non-specific pattern of autofluorescence seen in the negative control sample (D - ii) of skin. vWF and CD144 both appear to be more prominent on vascular structures in the dermis than liver blood vascular endothelial cells. All images were acquired in exactly the same manner, using the same camera exposure times, and post-capture analysis. All samples in this image underwent exactly the same staining procedure in the same experiment.

Supplementary Figure 3

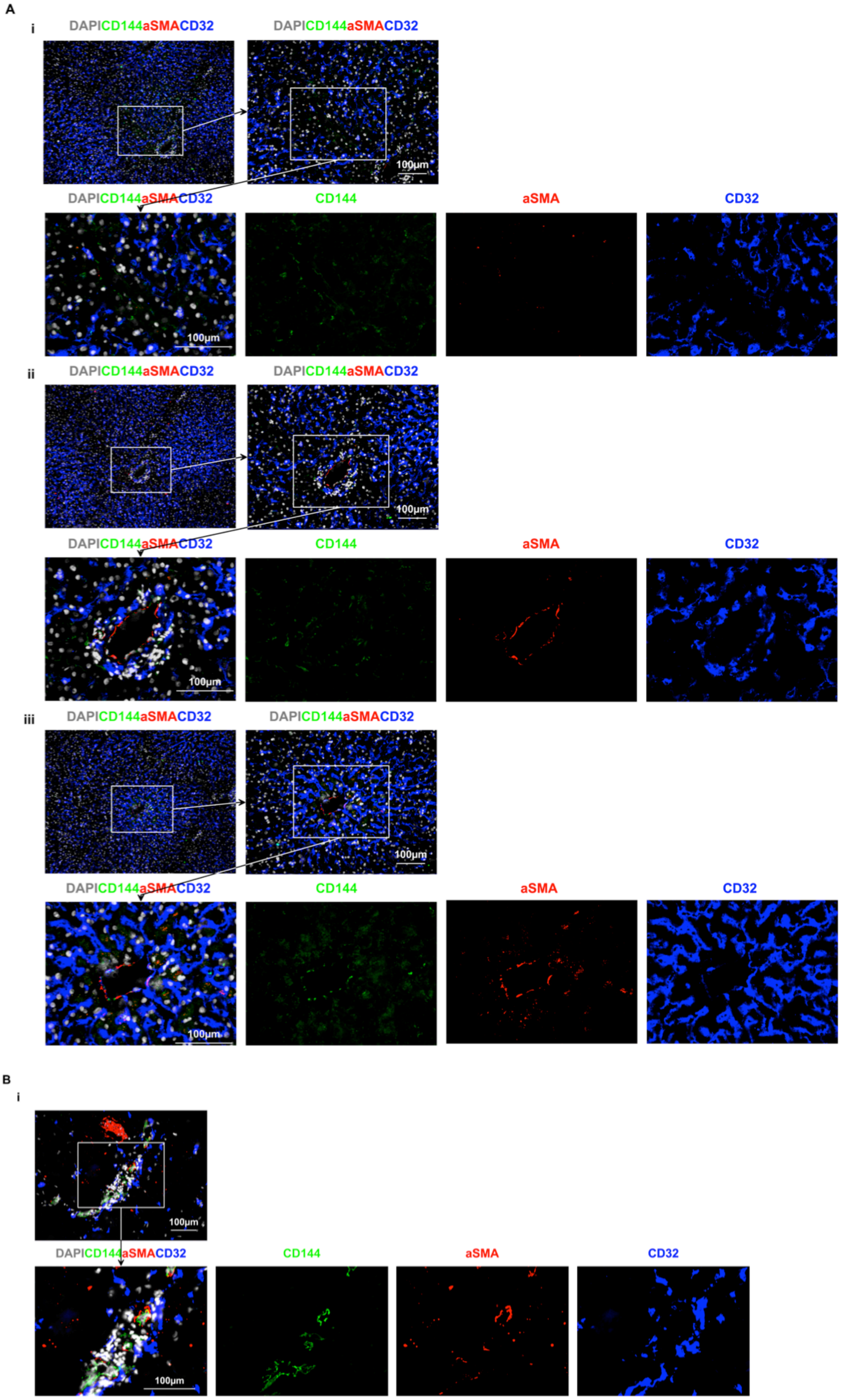

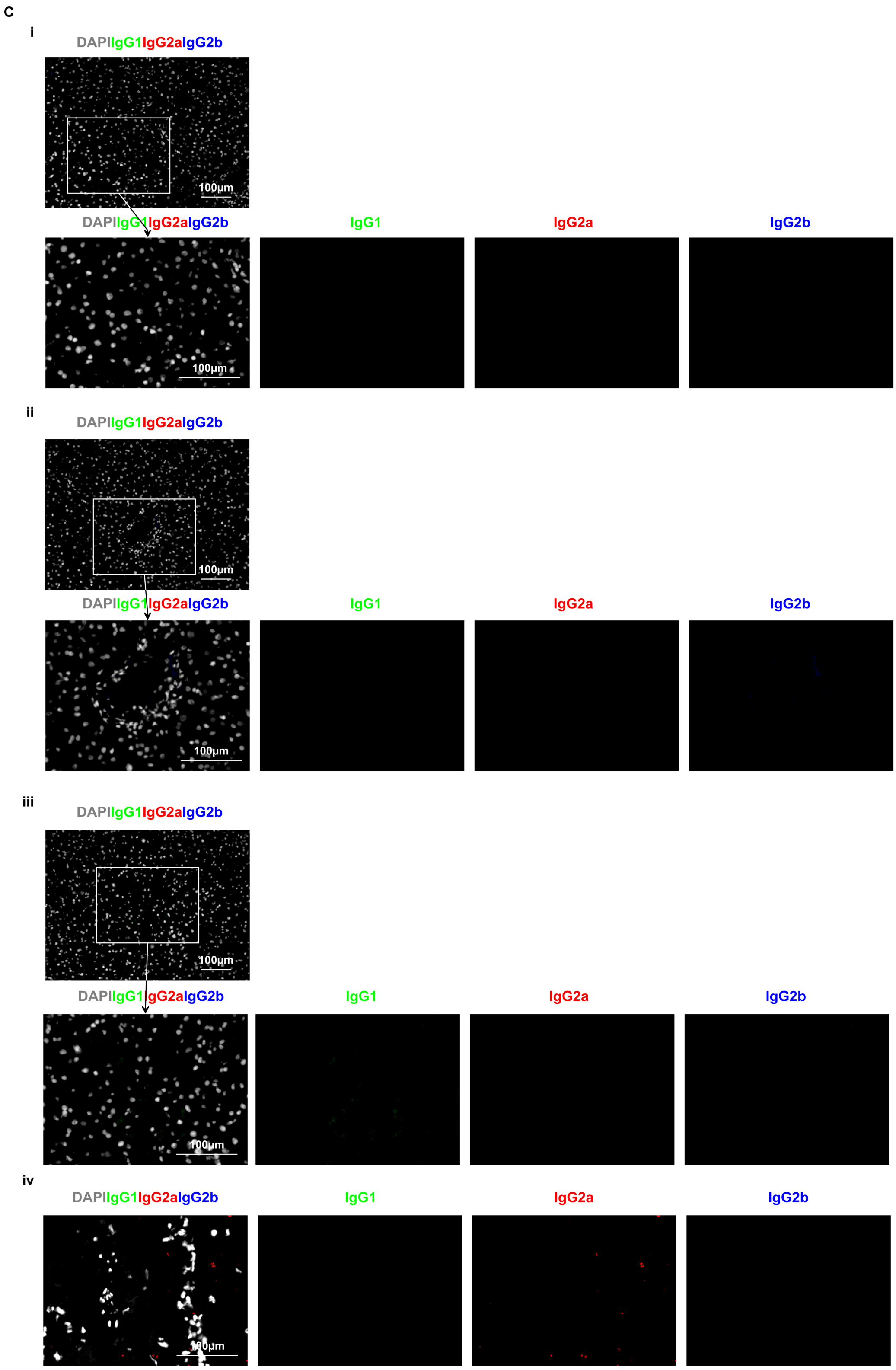

Supplementary Fig. 3

This figure presents images acquired following incubation with the IgG1 anti-CD144 isotype. (A) shows staining patterns characteristic of Z1(A – i), PT (A – ii), and CV (A- iii) areas. Similar to the IgG2a anti-CD144 isotype (Biolegend 348502

see Supplemantary Fig. 2) there is expression on CV and PT structures as well weak expression by cells in Z1, and inconsistent and variable expression throughout the lobule. The lobular expression does not follow a pattern and can only occasionally and inconsistently be seen on LSEC (identified through CD32 co-expression and as would be considered consistent with LSEC shape). This pattern of staining is distinct from the non-specific pattern of autofluorescence seen in the negative control samples of Z1 (C – i), PT(C - ii), and CV(C - ii) which did not undergo incubation with primary antibodies. Skin biopsy tissue was used as a positive control and is expressed by vascular endothelial cells in the dermis (B), this staining pattern is distinct from the non-specific pattern of autofluorescence seen in the negative control sample (C - iv) of skin. This appears to be more prominent on vascular structures in the dermis than liver blood vascular endothelial cells. All images were acquired in exactly the same manner, using the same camera exposure times, and post-capture analysis. All samples in this image underwent exactly the same staining procedure in the same experiment.

Supplementary Figure 4

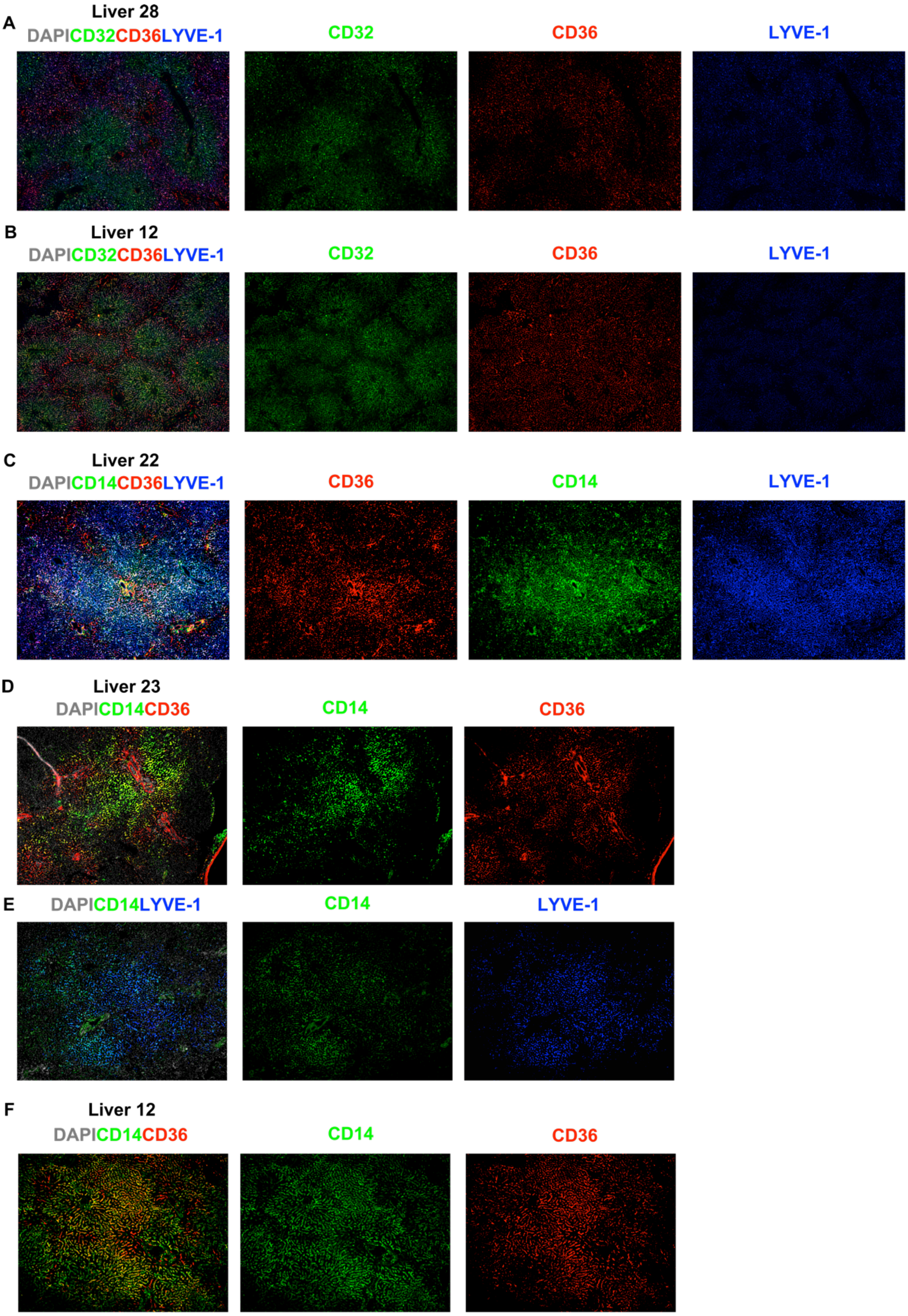

Supplementary Figure 4

The pattern of Type 1 and Type 2 LSEC was present in all livers assessed. This

figure shows more examples of each when assessing CD36(A, B, C , D, F), CD14 (C, E , F), CD32 (A, B) and Lyve 1(A, B, C, E). D and E are both Liver 23. This is supporting evidence to highlight that the findings presented with found in a minimum of 3 biological replicates, and all samples assessed.

Supplementary Tables

Table 1 Patient details

Patient	Age	Sex	Details	Organ
**1**	38	F	Resection of haematoma secondary to adenoma	Liver
**2**	29	M	Living Donor LT	Liver
**3**	21	M	Living Donor LT	Liver
**4**	21	F	Living Donor LT	Liver
**5**	53	F	Liver resection to remove cyst	Liver
**6**	50	F	Liver resection of cyst adenoma	Liver
**7**	46	F	Liver resection of adenoma	Liver
**8**	63	M	Liver Resection for haemangioma	Liver
**9**	56	F	Abdominoplasty	Skin

**10**	61	F	Breast reduction	Skin

Table 2 Antibodies used

Target	Species	Clone	Brand	Cat #	IF-Frozen	IF-Paraffin
**aSMA**	Mouse	1a4	Cellmarque	CM202M95	Y	Y
**CD13**	Rabbit	SP187	CellMarque	CM113R15	Y	Y
**CD14**	Rabbit	SP192	Abcam	ab183322	Y	Y
**CD14**	Mouse	MEM-18	Serotec	MCA2185	Y	N
**CD16**	Rabbit	SP175	Cellmarque	CM116R14	Y	Y
**CD31**	Mouse	JC70	CellMarque	CM131M94	Y	Y
**CD32**	Mouse	FUN-2	BioLegend	303201	Y	N
**CD34**	Rabbit	EP373Y	AbCam	ab81289	Y	Y
**CD36**	Mouse	185-1G2	AbCam	ab76521	Y	N
**CD45**	Mouse	F10-89-4	Abcam	ab30470	Y	Y
**CD54**	Mouse	LB-2	BD Biosciences	559047	Y	N
**CD64**	Mouse	10.1	BioLegend	305002	Y	N
**CD68**	Mouse	Y1/82A	BD Biosciences	556059	Y	N
**CD68**	Mouse	Kp-1	CellMarque	168M-94	N	Y
**CD105**	Mouse	MRQ-14	CellMarque	105M-14	Y	N
**CD144 (VE-cadherin)**	Mouse	BV9	Biolegend	348502	Y	N
**CD144 (VE-cadherin)**	Mouse	55-7H1	BD	555661	Y	N
**CD146**	Rabbit	EPR3208	Abcam	ab75769	Y	Y
**CD146**	Mouse	P1H12	BD Biosciences	550314	Y	Y
**CD206**	Mouse	5C11	AbCam	ab117644	Y	Y

**CD209/299***	Mouse	DCN46	BD Biosciences	551186	Y	N
**CD299**	Rabbit	Polyclonal	AbCam	ab58603	N	Y
**Cytokeratin 19**	Mouse	A53-B/A2	Biolegend	628502	Y	Y
**Cytokeratin 19**	Rabbit	EP72	MyBioSource	MBS370050	Y	Y
**Laminin 1+2**	Rabbit	Polyclonal	Abcam	ab7463	Y	Y
**LYVE-1**	Rabbit	Polyclonal	Abcam	ab36993	Y	Y
**vWF**	Mouse	2F2-AP	BD Biosciences	555849	Y	Y

Table 3 Number of biological replicates performed for each antigen presented.

**Antigen**	**Number of donors observed**
CD13	4
CD14	6
CD16	3
CD31	3
CD32	6
CD34	4
CD36	8
CD54	4
CD105	4
CD144 (VE-cadherin)	3
CD146	7
CD209/CD299/DC-SIGN	5
Alpha Smooth Muscle Actin	4
Cytokeratin 19	4
LYVE-1	6
Laminin 1+2	6
vWF	3

Table 4 - Findings comparing fluorescence in LSEC makers between zones.

Marker	Difference	SED	t	LSD	lwr	upr	p	Ratio	lwrRatio	uprRatio
CD14	0.1854	0.0384	4.8282	0.0789	0.1065	0.2643	0.0001	1.203699824	1.112377926	1.302518893
CD32	0.4516	0.0447	10.1132	0.0918	0.3598	0.5434	<0.0001	1.570823493	1.433042777	1.721851215
CD36	-0.1866	0.0555	-3.3621	0.1141	-0.3006	-0.0725	0.0024	0.82977558	0.740373863	0.930065747
LYVE1	0.6038	0.064	9.4412	0.1315	0.4724	0.7353	<0.0001	1.829056024	1.603838791	2.086107731

Difference = mean Intensity Z3 - mean Intensity zone Z1

SED = standard error of the difference between the means

t = Difference/SED

LSD = least significant difference is the minimum absolute difference between a pair of means in order for them to be considered statistically significant at the 0.05 level.

lwr = Difference – LSD (lower limit of 95% confidence interval for difference between pair of means)

upr = Difference + LSD (upper limit of 95% confidence interval for difference between pair of means)

Note: if range of values between lwr and upr includes zero, usually means not significant

ratio = exp(Difference) = ratio of median intensity of zone 3 to median intensity of zone 1

lwrRatio = exp(Difference – LSD) (lower limit of 95% confidence interval for ratio of medians)

uprRatio = exp(Difference + LSD) (upper limit of 95% confidence interval for ratio of medians)

Note: if range of values between lwrRatio and uprRatio includes 1, usually means not significant

Table 5 - Findings comparing intensity of fluorescence in PT and CV makers.

Marker	Difference	SED	t	LSD	lwr	upr	p	Ratio	lwrRatio	uprRatio
CD146	1.428	0.2275	6.2772	0.4879	0.9401	1.9159	0.0000	4.170350145	2.560237429	6.793049791

aSMA	0.6675	0.1958	3.4096	0.4199	0.2476	1.0874	0.0042	1.949357829	1.280947451	2.966551004
Laminin	1.1238	0.2519	4.4609	0.5403	0.5835	1.6641	0.0005	3.076522806	1.792300518	5.280918284

Difference = mean Intensity PT - mean Intensity zone CV

SED = standard error of the difference between the means

t = Difference/SED

LSD = least significant difference is the minimum absolute difference between a pair of means in order for them to be considered statistically significant at the 0.05 level.

lwr = Difference – LSD (lower limit of 95% confidence interval for difference between pair of means)

upr = Difference + LSD (upper limit of 95% confidence interval for difference between pair of means)

Note: if range of values between lwr and upr includes zero, usually means not significant

ratio = exp(Difference) = ratio of median intensity of PT to median intensity of CV

lwrRatio = exp(Difference – LSD) (lower limit of 95% confidence interval for ratio of medians)

uprRatio = exp(Difference + LSD) (upper limit of 95% confidence interval for ratio of medians)

Note: if range of values between lwrRatio and uprRatio includes 1, usually means not significant
